# Family Based Whole Exome Sequencing Reveals the Multifaceted Role of Notch Signaling in Congenital Heart Disease

**DOI:** 10.1371/journal.pgen.1006335

**Published:** 2016-10-19

**Authors:** Christoph Preuss, Melanie Capredon, Florian Wünnemann, Philippe Chetaille, Andrea Prince, Beatrice Godard, Severine Leclerc, Nara Sobreira, Hua Ling, Philip Awadalla, Maryse Thibeault, Paul Khairy, MIBAVA Leducq consortium, Mark E. Samuels, Gregor Andelfinger

**Affiliations:** 1 Cardiovascular Genetics, Department of Pediatrics, CHU Sainte-Justine, Université de Montréal, Montreal, Québec, Canada; 2 Faculty of Biology, University of Muenster, Muenster, Germany; 3 Department of Pediatrics, Centre Mère Enfants Soleil, Centre Hospitalier de l'Université (CHU) de Québec, Quebec City, Québec, Canada; 4 Omics-Ethics Research Group, Research Institute of Public Health, Université de Montréal, Montréal Québec, Canada; 5 McKusick Nathans Institute of Genetic Medicine, Johns Hopkins University School of Medicine, Baltimore, Maryland, United States of America; 6 Ontario Institute for Cancer Research, Toronto, Ontario, Canada; 7 Montreal Heart Institute, Université de Montréal, Montréal, Québec, Canada; 8 Membership of the MIBAVA Leducq consortium is provided in the Acknowledgements; 9 Centre de Recherche CHU Sainte Justine, Université de Montreal, Montréal, Québec, Canada; 10 Department of Medicine, Université de Montreal, Montréal, Québec, Canada; Georgia Institute of Technology, UNITED STATES

## Abstract

Left-ventricular outflow tract obstructions (LVOTO) encompass a wide spectrum of phenotypically heterogeneous heart malformations which frequently cluster in families. We performed family based whole-exome and targeted re-sequencing on 182 individuals from 51 families with multiple affected members. Central to our approach is the family unit which serves as a reference to identify causal genotype-phenotype correlations. Screening a multitude of 10 overlapping phenotypes revealed disease associated and co-segregating variants in 12 families. These rare or novel protein altering mutations cluster predominantly in genes (*NOTCH1*, *ARHGAP31*, *MAML1*, *SMARCA4*, *JARID2*, *JAG1*) along the Notch signaling cascade. This is in line with a significant enrichment (Wilcoxon, p< 0.05) of variants with a higher pathogenicity in the Notch signaling pathway in patients compared to controls. The significant enrichment of novel protein truncating and missense mutations in *NOTCH1* highlights the allelic and phenotypic heterogeneity in our pediatric cohort. We identified novel co-segregating pathogenic mutations in *NOTCH1* associated with left and right-sided cardiac malformations in three independent families with a total of 15 affected individuals. In summary, our results suggest that a small but highly pathogenic fraction of family specific mutations along the Notch cascade are a common cause of LVOTO.

## Author Summary

Left-ventricular outflow tract obstructions comprise a group of developmental heart disorders that are genetically and phenotypically heterogeneous, with no single gene accounting for the majority of cases. In order to identify mutations contributing to disease, we selected patients from 51 families with a history of congenital cardiac malformations. We interrogated the entire coding sequences of 106 patients and identified a small but highly pathogenic fraction of mutations that are likely to contribute to disease in 12 families (23.5%). Furthermore, we present a strategy for identifying candidate mutations based on familial segregation in a genetically heterogeneous disorder.

## Introduction

Left-ventricular outflow tract obstructions (LVOTO) comprise a group of cardiac malformations that restrict blood flow in the left portion of the heart. This heterogeneous subclass of cardiac malformations is commonly associated with aortic valve disease and early onset aortopathy, including severe stenosis and aortic dilation. These complications often result in a high disease burden later in life manifested by thoracic aortic aneurysm and valve replacement. Despite recurrent clustering of LVOTO traits among family members and a strong hereditary component [1,2], the underlying genetic cause of disease remains largely enigmatic [3]. Recent whole-exome sequencing and genotyping approaches revealed single nucleotide variants (SNVs) and copy number variations (CNVs) in genes that play an important role during the course of early outflow tract development and in chromatin modifications [4,5]. The list of known CHD disease genes is rapidly expanding and can be broadly defined by three functional groups: transcriptional regulators, cardiac structural proteins and signaling components. Disrupting mutations in critical factors regulating cardiac gene expression, such as *NKX2-5*, *TBX5* and members of the GATA gene family (*GATA4-GATA6*) have been identified in family based studies for congenital heart disease (CHD) [6–9]. In addition, rare missense mutations in structural proteins of the cardiac muscle, including *FLNA* and *MYH6* have been associated with single gene disorders and isolated cases of CHD [10]. However, the major fraction of patients with CHD cannot be solely explained by mutations in known disease genes [3]. Recent studies suggest that multiple genes in conserved signaling pathways mediating important cues for crucial processes during early embryonic development contribute to disease. Both familial and sporadic occurrences of LVOTO have been associated with mutations in genes of the Notch-signaling cascade [11,12]. Multifaceted Notch signaling plays a crucial role in cardiac cell fate regulation and orchestrates the morphogenesis of cardiac chambers and valves [13–15]. A recent study has suggested that *NOTCH1* haploinsufficiency alters specific gene networks affecting valve development and osteogenic factors which in turn result in aortic valve disease [16]. This is consistent with the identification of the *NOTCH1* ligands *JAG1* and *DLL4* which have been shown to cause Alagille syndrome and aortic valve disease in human and animal models [17–19]. However, the high functional redundancy of the Notch signaling cascade and the multitude of overlapping phenotypes between interacting genes suggest a certain level of locus heterogeneity associated with disease. Here, we report our results from a family based approach that aims to decipher the underlying genetic heterogeneity of LVOTO traits in a pediatric cohort including 182 patients from 51 families of French Canadian origin. Central to our approach is the family unit which serves as a reference to identify causal genotype-phenotype correlation.

## Results

### Familial based whole-exome sequencing of CHD

**Fig 1:**
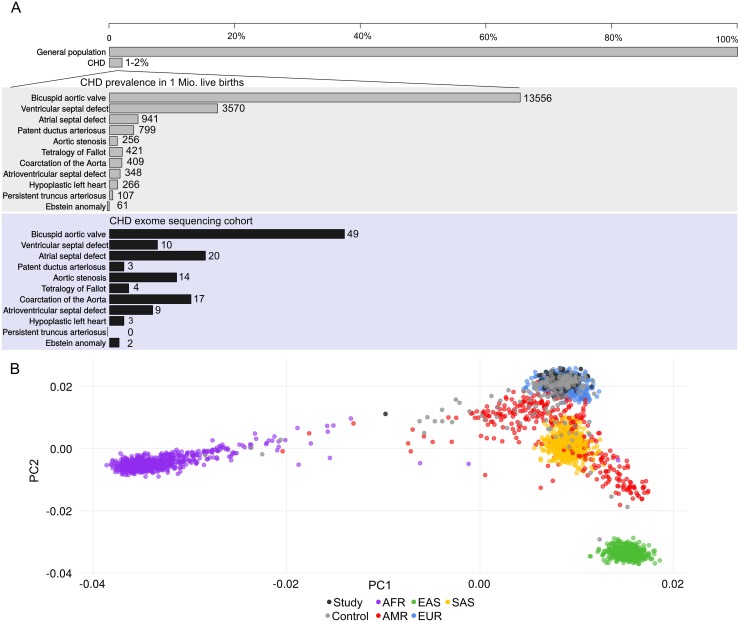
Overview of the study cohort. **(A)** Prevalence and phenotypes associated with CHD in the general population and in the presented cohort. The numbers highlighted in grey indicate the prevalence of CHD phenotypes in 1 million live births according to Fahed et al. [3]. The graph highlighted in blue represents the distribution of overlapping CHD phenotypes in our cohort, which show a similar distribution of what is expected from the general CHD population. **(B)** Principal component analysis of individuals from the French Canadian pediatric cohort presented in this study with 2504 individuals from the 1000 Genomes phase 3 release for the five human super populations (AFR: purple, AMR: red, EUR: blue, EAS:green, SAS:yellow). Study subjects (black dots) and controls (grey dots) predominantly cluster with individuals of European descent (blue dots).

**Fig 2:**
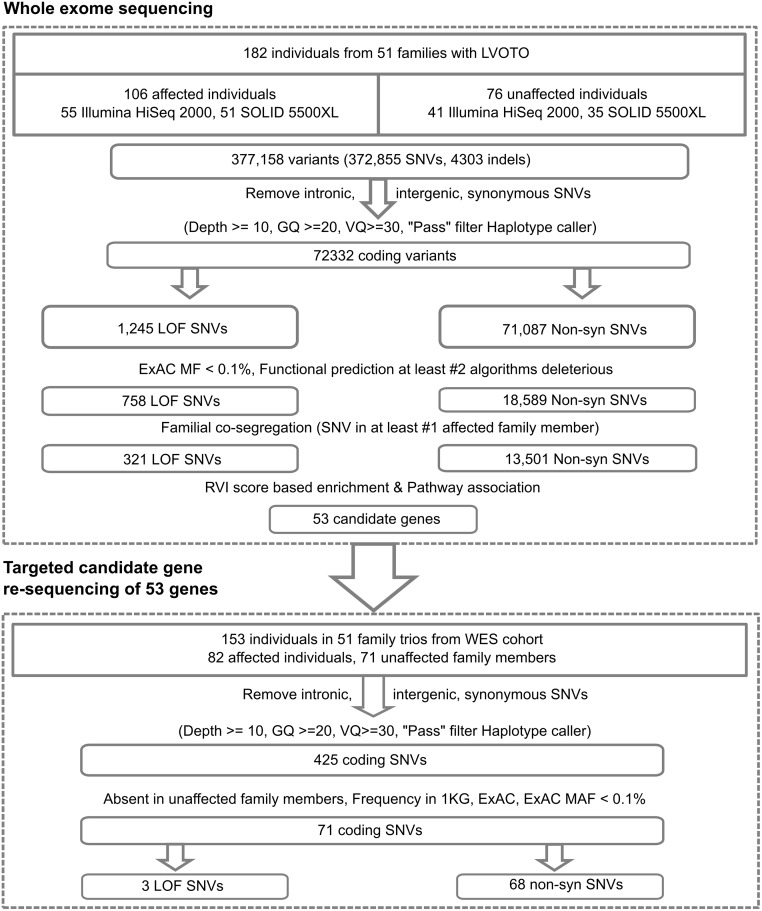
Variant filtering criteria and candidate gene prioritization. Variants were called using the GATK pipeline as outlined in the method section. Several stringent quality and allele filtering thresholds were applied prior to filtering for allele frequency and co-segregation among family members. Annotation and filtering of coding SNVs was performed using the ANNOVAR pipeline for RefSeq gene annotations [65].

Whole-exome sequencing was performed in 182 individuals from 51 families with a strong heritable history of congenital heart disease (CHD) on the SOLiD 5000xl and Illumina HiSeq2000 platform (Fig 1a). The average read depth for the targeted platforms was 82x with 78% of targeted regions covered at greater than 20x. The ethnicity of our cohort was evaluated using principal component analysis and revealed that all except 1 patient, who was removed from subsequent analysis, clustered closely with individuals of European descent (Fig 1b) [20]. We evaluated the overall burden of co-segregating disease mutations for protein coding genes in our families instead of performing family-based association analysis due to the modest sample size and phenotypic heterogeneity of our cohort [21]. Among all 182 samples, 72,332 potential deleterious coding SNVs were identified of which 1245 were loss-of-function SNVs, including splice site disrupting variants, stop-gain mutations, frameshift indels and 71087 non-synonymous SNVs (Fig 2). In order to identify candidate deleterious variants, we applied four prediction algorithms for variant pathogenicity (SIFT, Poly-Phen-2, Mutation Taster, Mutation Assessor) and the CADD method [22]. All non-synonymous variants predicted to be damaging by at least two algorithms were included for further downstream analysis. We then filtered for rare variants, based on minor allele frequencies from the Exome Aggregation consortium (ExAC) ExAC Total MAF < 0.1%) and for variants which co-segregated with disease within families. This family-based approach that includes disease-segregation among multiple affected as filtering criteria, significantly reduced the number of potential pathogenic variants per patient in our family cohort (S1 Fig). As a next step, we prioritized candidate genes among the remaining 321 loss of function and 13,501 non-synonymous variants under the assumption that an excess of rare deleterious variants (rdSNVs) in genes intolerant to deleterious mutations would be more likely to affect gene function and contribute to disease in our cohort.

### Enrichment of deleterious variants in highly conserved genes

**Fig 3:**
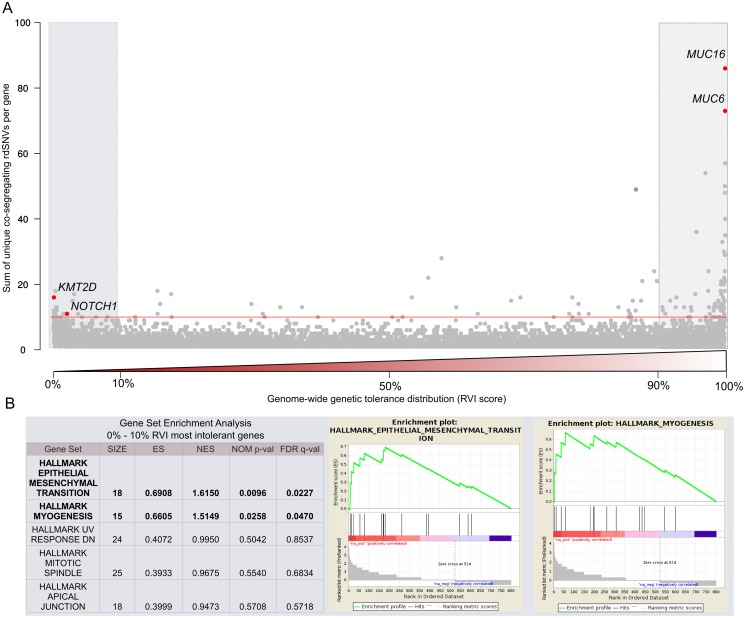
Candidate gene prioritization in family based whole exome sequencing. **(A)** Cumulative sum of unique and rare (ExAC <0.1%) deleterious variants co-segregating with disease for each gene in the whole exome dataset against the genome-wide RVI score percentiles. The red line indicates a transformed z-score thresold cutoff (p-value < 0.01), highlighting genes which have acquired more than 10 rare deleterious variants in the whole-exome dataset (z-score > 2). Candidate genes *NOTCH1* and *KMT2D* in the top 10% of intolerant genes as well as likely false positive *MUC16* and *MUC6* in the top 10% of tolerant genes, are highlighted in red. **(B)** Pathway enrichment of genes among the 10^%^ RVI percentile which harbor two or more rdSNVs in 106 whole-exome sequenced probands. Ranked Gene Set Enrichment analysis was performed on z-score based gene ranking with the weighted parameter and 1000 permutations for 50 BROAD derived hallmark gene sets.

To reduce the noise of our enrichment analysis, we further selected genes based on the Residual Variation Intolerance Score (RVIS) statistic [23]. Fig 3a highlights the enrichment of co-segregating rdSNVs in genes towards the lower and higher end of the genome-wide genic intolerance distribution based on their RVI scores. This unbiased enrichment approach for rare variants segregating with disease revealed 14 candidate genes showing an enrichment of rdSNVs (z-scores > 2) in our cohort among the top 10% most intolerant genes genome-wide (Fig 3a, S1 Table). Among these are two promising candidate genes, *NOTCH1* and *KMT2D* harboring rdSNVS co-segregating with disease in multiple families. *NOTCH1* is associated with aortic valve disease [11,12] whereas variants in *KMT2D* cause the pediatric disorder Kabuki syndrome with multiple congenital malformations including aortic valve disease [24–26]. Overall, we observed a significant excess (p< 0.01, Wilcoxon) of co-segregating variants among highly conserved genes (RVIS < 10^th^ percentile) in our data set (S2 Fig). We used the Gene Set Enrichment Analysis (GSEA) to investigate the potential biological relevance of a higher genetic burden in conserved genes for 50 hallmark gene sets from the Molecular Signatures Database [27–29]. Hallmark gene sets represent well-defined biological states that display coherent expression and were considered significantly enriched with a FDR multiple test correction p value of < 0.05. We found significant enrichment in the two hallmark gene sets, “myogenesis” and “epithelial-mesenchymal transition” (Fig 3b). The highly conserved genes related to “myogenesis” and “epithelial-mesenchymal transition” are likely to play a role in disease etiology due to their crucial role during cardiac development [30,31]. Contrary to the enrichment of co-segregating rdSNVs in genes intolerant to deleterious variants, an excess of rdSNVs was observed in genes with a high genome-wide tolerance of common variants (RVIS > 90^th^ percentile) (S2 Fig). This is to be expected, since genes on this extreme end of the RVIS distribution include frequently mutated loci such as *MUC6* and *MUC16* (Fig 3a) that accumulated an excess of low frequency variants due to their length and exon number which makes them unlikely contributors to CHD [32–34]. Finally, mutational burden testing was performed on highly conserved candidate genes (RVIS < 10^th^ percentile) in order to determine the contribution of individual candidate genes to disease in our cohort, based on collapsing co-segregating rdSNVs into a single gene score. We compared 106 affected individuals from our family cohort with 193 non-consanguineous population controls. The control samples sequenced at the Baylor-Hopkins Center for Mendelian Genomics were selected from the Mendel cohort without a history of cardiovascular disease and cluster predominantly with our samples of French Canadian origin (Fig 2b) [35]. While we did not observe a significant enrichment of rdSNVs in a single candidate gene, testing for novel mutations revealed a significant burden in *NOTCH1* (novel alleles 0/193, novel alleles in LVOTO cohort 3/106, p = 0.0462, Fisher exact test one tailed). This is in line with an earlier association of *NOTCH1* with bicuspid aortic valve disease highlighting a significant excess of private mutations in the locus [36]. After gene prioritization, we retained 53 candidate genes based on the following criteria: harboring co-segregating variants observed in at least one family, pathway association with the hallmark pathways “myogenesis”, “epithelial-mesenchymal transition” and “chromatin modification” for downstream analysis (S3 Fig)

### Detailed analysis of families with NOTCH1 mutations

**Fig 4:**
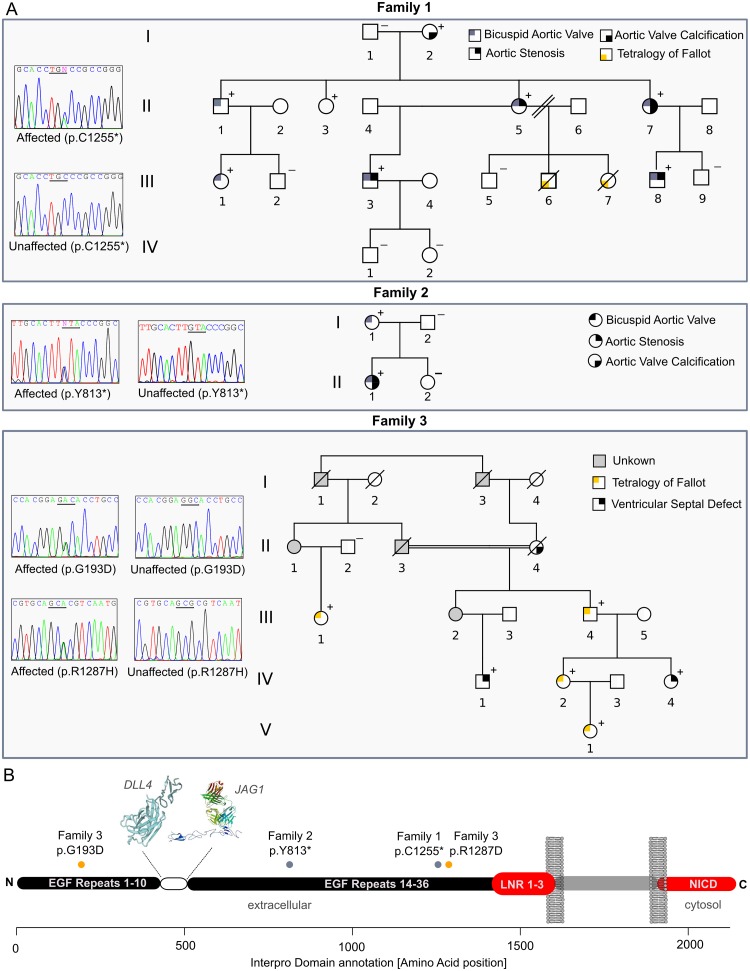
*NOTCH1* mutations in three independent families. **(A)** Pedigrees of families harboring high impact *NOTCH1* mutations. Colors represent the different phenotype associations. The (+/-) symbols indicate mutation carrier status. **(B)** Schematic representation of the *NOTCH1* locus. Clustering of protein truncating mutations is observed in the extracellular part of the protein in the EGF domains 1–36.

In the next step of our family-based approach, we performed a detailed analysis of affected family members in three families sharing novel and deleterious mutations in *NOTCH1* that are likely to contribute to disease (Fig 4a, S2 Table). Phenotypic analysis of affected family members revealed the absence of syndromic disease and indicated a high rate of cardiac valve anomalies and vascular obstructions (Table 1). Two novel *NOTCH1* nonsense mutations in two independent families were identified, both of which are located in the extracellular domain of the protein that has been associated with aortic valve disease [11] (Fig 4b). Both stop-gain mutations (Family 1: c.C3765A:p.C1255*; Family 2: c.C2439G:p.Y813*) are located in the N-terminal EGF domain repeats 14–36 of NOTCH1, potentially leaving the ligand binding site of the protein intact if escaping nonsense-mediated decay. The affected mother in family 1 (Family1, II-5) (Fig 4) had a bicuspid aortic valve and severe aortic stenosis. She is a mutation (c.C3765A:p.C1255*) carrier and had been remarried after conceiving two siblings who died of Tetralogy of Fallot (TOF) shortly after birth. Unfortunately, DNA was not available for genetic characterization of the variant in these patients. The affected half-brother had valve dysfunction and a bicuspid aortic valve, which indicates a strong penetrance of the Mendelian variant within this family. The inheritance patterns of valve disease in family 1 highlight the importance of different genetic backgrounds for the expressivity of the associated phenotype. While members of family 1 and 2 had primarily left-sided cardiac malformations, affected members in family 3 suffered primarily from right-sided cardiac defects. We identified two highly conserved *NOTCH1* missense mutations in cis in family 3, of which one is novel (c.G578A:p.G193D) and one is rare (7/73804 ExAC alleles, c.G3860A:p.R1287H) in the general population. Both mutations segregate with disease on the same haplotype with TOF or ventricular septal defects in five affected family members. The novel mutation c.G578A:p.G193D is predicted to be highly deleterious by all major algorithms (SIFT score = 0, Polyphen-2 score = 1, CADD score = 32) [22,37,38]. Both mutations resided in the EGF repeat domains 4 and 33 and are located in the extracellular *NOTCH1* domain which mediates ligand binding to JAG1 and DLL4 [39]. In order to gain additional evidence of disease association in family 3, we performed linkage analysis on the extended family (9 individuals). The analysis supported the whole-exome based association with *NOTCH1* with a genome-wide linkage signal of LOD > 1.5 at four genomic loci (chr1, chr9, chr10 and chr15) (S4a Fig). When only considering rare co-segregating variants under the linkage peaks, solely *NOTCH1* remains as a potential candidate gene for this family (S4b Fig). While protein disrupting mutations in NOTCH1 are likely to contribute to disease, interpreting the role of additional deleterious mutations in genes of uncertain function that may act epistatically remains challenging in a family based context (S2 Table). Apart from the highly pathogenic variants in *NOTCH1*, we identified a family trio with a private co-segregating nonsense mutation in *ARHGAP31* (Family IV: c.A4222T:p.K1408*) in our cohort (S5 Fig, S2 Table). *ARHGAP31*, a Rho GTPase and specific regulator of the RAC1/CDC42 axis has been associated with valve disease and is strongly and specifically expressed in the course of early heart development in mouse [40] and in chicken (S6 Fig). The father, carrying the loss of function variant in *ARHGAP31* in family 4 (Family 4, I-1) (S5 Fig) was diagnosed with aortic valve disease, manifested as aortic regurgitation, bicuspid aortic valve and a slight dilation of the ascending aorta. His son had been diagnosed with aortic stenosis early in life, associated with a bicuspid aortic valve, and developed a dilation of the ascending aorta later in life. Both patients showed no history of syndromic disease. Notably, *NOTCH1* and *ARHGAP31* mutation carriers overlap in their phenotypic spectrum, showing a clustering of valvular phenotypes and aortopathies. Furthermore, *ARHGAP31* and *NOTCH1* have both been associated with Adams-Oliver syndrome (OMIM#100300, OMIM#616028), which is associated with severe cardiac malformations such as valvular defects and TOF [40,41]. These findings suggest a certain degree of locus heterogeneity in our cohort.

**Table 1:** Clinical features of three families carrying pathogenic *NOTCH1* mutations.

Individual (Family-Generation-Individual)	Mutation Status	Clinical Features		
		Cardiac defects	Vascular defects	Other Remarks
1-I-2	+	Aortic valve sclerosis	Aortic regurgitation	
1-II-1	+	Bicuspid aortic valve, Aortic Valve calcification	Aortic stenosis	Ross procedure
1-II-5	+	Bicuspid aortic valve	Aortic stenosis, Dilation of the ascending aorta	Valve replacement
1-II-7	+	Bicuspid aortic valve		
1-III-1	+	Bicuspid aortic valve		
1-III-3	+	Bicuspid aortic valve	Aortic stenosis	
1-III-6	noDNA	Tetralogy of Fallot		Died shortly after birth
1-III-7	noDNA	Tetralogy of Fallot		Died shortly after birth
1-III-8	+	Bicuspid aortic valve	Aortic stenosis, Dilation of the ascending aorta	Valvuloplasty with balloon catheter
2-I-1	+	Bicuspid aortic valve		
2-II-1	+	Bicuspid aortic valve	Aortic Stenosis, Dilation of the ascending aorta	Valvuloplasty with balloon catheter
3-III-1	+	Tetralogy of Fallot		
3-III-4	+	Tetralogy of Fallot		
3-IV-1	+	Ventricular septal defect		
3-IV-2	+	Tetralogy of Fallot		
3-IV-4	+	Ventricular septal defect		
3-V-1	+	Tetralogy of Fallot		

### Targeted re-sequencing of candidate genes associated with CHD

**Fig 5:**
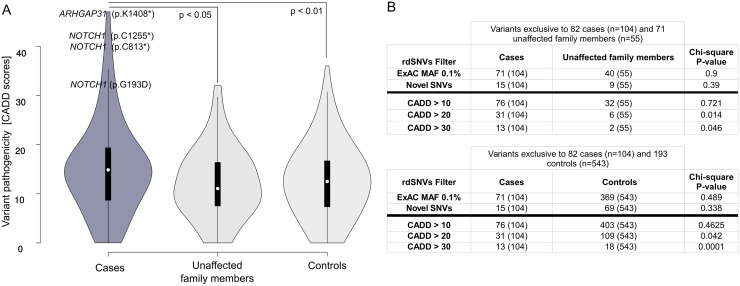
Association between variant pathogenicity and allele frequencies. **(A)** Variant pathogenicity based on scaled CADD scores is significantly higher in familial cases compared to unaffected family members (p < 0.05, Wilcoxon-rank sum test) and controls from the Mendel dataset (p < 0.01, Wilcoxon-rank sum test). **(B)** This enrichment of variant pathogenicity is due to a small fraction of variants exclusively observed in cases exceeding CADD scores >20 (p < 0.05, chi-square test). In contrast, there was no significant enrichment observed for rare (ExAC MAF < 0.1%) or novel deleterious variants in cases compared to unaffected family members or population controls.

To test the hypothesis that multiple candidate loci contribute to disease and eliminate gene coverage biases in our 53 previously identified candidate genes, we performed Nimblegen targeted re-sequencing for 153 patients from our initial whole-exome sequencing cohort in a trio design which included 82 affected individuals and 71 unaffected family members (Fig 1). We achieved an average coverage of 71.4x and 99% of the target exons were covered at 20x. Principal component analysis of our candidate genes against the ExAC dataset revealed that several genes including *MYH6*, *KMT2D*, *EP300* and *EP400* show an enrichment of rare private mutations despite their high intolerance to common mutations (RVIS: *MYH6* = -2.78, *KMT2D* = -5.29) in the ExAC cohort. This is also reflected by an enrichment of rare benign mutations in these intrinsically variable genes (S7 Fig) based on ClinVar annotation. Despite the significant reduction of benign mutations by applying stringent allele filtering thresholds (p = 0.021; Fisher exact test), it remains a challenging task to discriminate between causative and non-causative missense mutations in common and often asymptomatic diseases of the aortic valve, such as BAV. For this reason, we used the recently developed Combined Annotation Dependent Depletion (CADD) framework to characterize and rank co-segregating mutations in our candidate genes [22]. This framework provides a genome-wide estimate of protein altering mutations and can be used as a meta-annotation tool to rank variants according to their pathogenicity scores. Based on the unbiased CADD score statistics, we observed a significant (p< 0.05, Wilcoxon) enrichment of deleterious mutations in patients compared to unaffected family members and 193 population controls from the Mendel dataset (Fig 5a). This enrichment in variant pathogenicity in our family cohort is due to a small fraction of variants exclusively observed in patients exceeding CADD scores >20 and ranking among the top 1% most pathogenic mutations genome-wide (p < 0.05, chi-square, Fig 5b). In contrast to the enrichment of pathogenic variants in patients, we did not observe an excess of rare or novel variants in cases when compared to controls (p > 0.05, chi-square, Fig 5b). These results are consistent with the notion that natural selection removes rare protein altering mutations from the general population and implies that only a small but highly pathogenic fraction of familial transmitted mutations contributes to disease in our cohort.

### Excess of inherited missense variants along the NOTCH signaling axis

To assess whether highly ranked pathogenic mutations are enriched in disease relevant gene sets, we compared their pathogenic burden for 50 hallmark gene sets in patients and controls using the Wilcoxon rank sum statistic. We found a significant enrichment of CADD scores in members of the Notch signaling and the previously highlighted myogenesis gene sets (p < 0.05, FDR adjusted Wilcoxon) (S3 Table). This enrichment was due to mutations residing in genes related to both pathways, including *NOTCH1*, *MAML1*, *JAG1*, *MYH6*, *ARHGAP31* and *EPHB4* with CADD scores > 20. Finally, by ranking mutations based on their predicted pathogenicity we were able to exclude mutations in intrinsically variable genes and identified the small but highly pathogenic fraction of transmitted missense mutations that are likely to contribute to CHD in 12 families of our 51 families (S3 Table). Notably, our highest ranked disease-segregating mutations clustered predominantly in genes along the Notch signaling axis (Table 2). We identified two families with mutations in *MAML1*, a transcriptional co-activator of *NOTCH1* involved in outflow tract defects [42,43]. Both rare mutations with CADD scores > 20 (c.G2129A:p.R710Q; c.G913A:p.A305T) segregated with aortic valve disease in five affected patients in both families, including a distantly related cousin. In addition, a family trio with a private mutation (c.G1540A:p.G959A) in *JARID2* was identified which segregated with bicuspid aortic valve in the index case and his father. *JARID2* has been reported to cause outflow tract malformations in a mouse model and regulates *NOTCH1* expression via histone modifications in the developing heart [44]. This is in line with the identification of a patient with an overlapping phenotype and a compound heterozygous mutation (maternal: c.C3830T:p.P1277L/paternal: T4211G:p.V1404G) in the Notch signaling repressor *SMARCA4* (BRG1). This analysis is however insufficient to conclusively assess novel gene associations with CHD based on family association alone, especially in the light of emerging evidence supporting a role of deleterious mutations inherited from unaffected family members [45].

**Table 2:** Candidate genes in familial LVOTO cluster predominantly along the Notch signaling axis.

Gene	OMIM Disease Association	Phenotype in exome cohort	Mutations in exome cohort	Family ID
*NOTCH1*	Adams-Oliver Syndrome, Aortic valve disease	Bicuspid aortic valve, Dilation of the ascending aorta, Tetralogy of Fallot	C1255*	Family 1
			Y813*	Family 2
			G193D/R1287H	Family 3
*ARHGAP31*	Adams-Oliver Syndrome	Bicuspid aortic valve, Severe aortic stenosis	K1408*	Family 4
*MYH6*	Dilated cardiomyopathy, Atrial septal defects	Hypoplastic left heart, Bicuspid aortic valve, Atrial septal defect, Aortic stenosis	M494I/E1885K (compound het)	Family 5
			T1379M	Family 6
*MAML1*	/	Bicuspid aortic valve, Severe stenosis	R710Q	Family 7
			A305T	Family 8
*EPHB4*	/	Severe pulmonary stenosis, Tetralogy of Fallot, Ventricular septal defects	P327L	Family 9
*JARID2*	/	Bicuspid aortic valve, Dilation aorta	G959A	Family 10
*JAG1*	Alagille Syndrome, Tetralogy of Fallot	Valve sclerosis, Aortic valve replacement, Severe aortic stenosis	P4L	Family 11
*SMARCA4 (BRG1)*	/	Bicuspid aortic valve, Dilation of the ascending aorta	P1277L/V1404G (compound het)	Family 12

### Sensitized genetic background in a family with pleiotropic CHD

The CADD based pathogenic ranking revealed a compound heterozygous mutation in *MYH6* in a family with pleiotropic congenital heart disease (S8 Fig). The index case in Family 5 (Family 5, III-3) recessive for rare maternal and paternal *MYH6* mutations (maternal:c.G5653A:p.E1885K/paternal: c.G1482A:p.M494I) died shortly after birth of hypoplastic left heart syndrome. This is consistent with a previously described disease association of recessive *MYH6* mutations in two patients with HLHs [46]. Notably, four affected family members in Family 5 are carriers of the paternal mutation that co-segregated in incomplete penetrance with aortopathy, septal defects and bicuspid aortic valve. This finding overlaps with a study, reporting incomplete penetrant autosomal dominant *MYH6* mutations in a family with an identical clustering of LVOTO subtypes [47]. This instance highlights the complexity of assessing causal genotype-phenotype correlations in families with CHD where the deleterious missense mutation with a higher pathogenicity in the index case (maternal CADD = 34/paternal CADD = 17.7) is inherited from the maternal branch of the family without a prevalent history of disease (S8 Fig).

## Discussion

In this study, we performed whole-exome and targeted candidate gene sequencing for a cohort of families with LVOTO in order to dissect the underlying genetic architecture of CHD. We identified a clustering of rare co-segregating mutations in highly conserved genes along the Notch signaling axis.

### NOTCH1 mutations in LVOTO

While both gain and loss of function mutations have been reported for *NOTCH1* [11,12,48], identification of families with multiple affected members carrying novel nonsense mutations remains rare in the context of congenital heart disease. In our cohort, we observed a mutational clustering in the extracellular NOTCH1 terminus in three families with 15 affected mutation carriers. This supports the notion that disruption of the cytosolic domain, but not the ligand binding part of the protein contributes to valve disease. Our findings are consistent with recent studies highlighting that the disruption of *NOTCH1* downstream signaling can affect epithelial-mesenchymal transition in the course of outflow tract development [30,49]. During this developmental process, that is enriched for rare deleterious mutations in our LVOTO cohort, endocardial cells detach to become a migratory mesenchyme which in turn is crucial for the proper formation of cardiac valves [30]. Notably, we did not observe a stringent genotype-phenotype correlation with *NOTCH1* deficiency and LVOTO subtypes across families in our cohort. The presence of right-sided outflow tract obstructions in families 1 and 3 indicates a degree of phenotypic heterogeneity for this locus. This is consistent with multiple disease associations of *NOTCH1* with right-sided lesions reporting that up to 18% of *NOTCH1* mutation carriers show right sided cardiac malformations [11,50]. The phenotypic expansion in our family cohort is intriguing due to the previous association of *NOTCH1 and ARHGAP31* with Adams-Oliver syndrome (AOS) [40,41]. When analyzing the molecular pathways disrupted in AOS and LVOTO, it seems likely that the specific CDC42/RAC1 regulator *ARHGAP31*, which is highly expressed in the developing mouse heart [40], and the Notch pathway converge in a common signaling route that is critical for outflow tract development. Notably we did not observe characteristics of Adams-Oliver syndrome in patients with *NOTCH1* nonsense mutations but one incidence of aortic valve disease.

### Enrichment of deleterious mutations along the NOTCH signaling cascade

Furthermore, chromatin remodeling is an important mechanism in neural crest cells in the process of valve cushion formation and required for outflow tract formation. This process is mediated by several of the candidate genes along the Notch signaling axis identified in this study including SMARCA4 JARID2 and MAML1. We speculate that the disruption of chromatin modifiers, such as *MAML1*, which functions as a transcriptional co-factor for NOTCH1, might predispose patients for the observed phenotype of outflow tract defects in our cohort. Targeted disruption of Notch downstream signaling in neural crest cells by dominant negative MAML1 (DNMAML) results in similar outflow tract malformations in an animal model of disease [43]. This is in line with the tissue specific role of transcriptional Notch repressors *JARID2* and *SMARCA4* (BRG1). Depletion of *Brg1* in endothelial cells frequently results in bicuspid aortic valve in mice and endothelial *JARID2* is required for normal cardiac development [44,51,52]. These findings support the role of locus heterogeneity in aortic valve disease where disruption of functionally related members of the same pathway can result in similar phenotypes in mice and men. This is emphasized by the identification of a highly pathogenic and rare disease segregating variant in *EPHB4*. In a mouse model, Ephb4 loss of function phenocopies the effect of *Notch1* mutations resulting in similar aortic malformations [53].

### Sensitized genetic background in CHD

Our study highlights that familial segregation and weighing a variant in favor of pathogenicity can serve as an important resource for discriminating potential disease causing variants in CHD in the absence of large sample sizes. Stringent allele filtering thresholds (ExAC < 0.1% MAF) and CADD score based pathogenicity ranking (scaled CADD scores > 15) are particularly useful in analyzing intrinsically variable genes such as *KMT2D* and *MYH6*. Such filtering allowed us to identify two recessive *MYH6* mutations in a family with pleiotropic cardiac malformations including HLHs, septal defects and aortopathy. Dominant *MYH6* mutations are known to have a variable penetrance and despite the absence of MYH6 expression in the outflow tract, impaired vascular function and decreased blood flow have been reported in an animal model [54]. This highlights how autosomal dominant transmitted missense mutations may create a sensitized genetic background which predisposes a family member to CHD despite being completely penetrant. The presence of a second mutation in the same locus or environmental perturbations would then be required to reach a disease state. This is likely the situation in the index patient with HLHs in family 5 (Family 5, III-3) (S5a Fig), where the maternal *MYH6* mutation with a higher pathogenicity is inherited from the branch of the family without a prevalent history of CHD.

### Limitations and conclusions

While we observed an enrichment of deleterious mutations in highly conserved genes along the multifaceted NOTCH signaling cascade, our study has limitations. First, none of our candidate loci showed a genome wide significant association with disease. This might be due to the sample size of our cohort which provides limited statistical power, the differences in sequencing enrichment kits and sequencing platforms used or more importantly the underlying complex genetic architecture of CHD.

In addition, environmental factors, such as aortic flow, or differences in the genetic background of family members were not considered in our study design. However, these factors are likely to contribute to the phenotypic heterogeneity and incomplete penetrance observed in families with CHD. Further functional assessment of the identified mutations in multiple loci, that may act epistatically, will be needed to unravel their role during the course of disease. Taken together, our study provides a broader insight in the genetic and phenotypic heterogenic landscape of CHD. We identified co-segregating mutations that are likely to contribute to disease in 12 of our 51 families (23.5%) in multiple loci, which is in line with several recent studies highlighting oligogenic inheritance patterns of non-syndromic CHD in families [45,55–57]. While the number of CHD associated genes increases with each exome study, there is also a growing body of evidence that these genes converge in a complex, yet discrete network driving outflow tract and vascular development [16,58,59].

## Material and Methods

### Patients

Families were recruited at CHU-Sainte Justine hospital within the French Canadian population. Participants were evaluated by clinical examination, standard 12 lead electrocardiography measurements as well as two-dimensional echocardiography. Written and informed consent was obtained from all patients or from parents in the case of pediatric patients. The study was approved by the CHU-Sainte Justine ethical board commission. The 193 unrelated control samples used for the burden test were selected among 865 samples from the Baylor-Hopkins Center for Mendelian Genomics (BHCMG) that were not from consanguineous family, of North American descent, had no known cardiovascular phenotypes and are unrelated [35].

### Whole-exome and targeted sequencing

DNA was extracted from peripheral blood using the Qiagen Gentra Puregene blood kit (QIAGEN, Toronto, Canada). 96 samples (55 affected individuals) were sequenced using standard library preparation protocols with the Agilent SureSelect 50Mb exome enrichment kit and subsequently subjected to 150 base pair, paired-end sequencing on the Illumina HiSeq2000 platform at the McGill Genome Center. Post-quality control reads were aligned to the reference human genome version 19 (hg19) using bwa [60]. A total of 86 samples (51 affected individuals) were sequenced using the TargetSeq enrichment kit developed for the SOLiD5500xl sequencing platform at CHU-Sainte Justine hospital. Sequencing was performed using bar coded multiplex runs of twelve samples on eight flowchips. Mapping was performed using LifeScopeTM 2.0 Genomic Analysis Software, on the hg19 genome builds. Nimblegene target re-sequencing was performed using a custom designed SeqCap panel for candidate genes and sequenced on the Illumina HiSeq2000 platform. Aligned reads were subsequently processed using Picard (http://picard.sourceforge.net) duplicate removal and Genome Analysis Toolkit (GATK v3.2), INDEL realignment, base quality score recalibration and SNP and INDEL discovery using Haplotypecaller according to GATK best practices [61]. The 193 controls from the Mendel Genomics cohort were sequenced on the Illumina HiSeq2000 platform and aligned using the GATK pipeline as described elsewhere [62].

### Variant prioritization and quality control

Variants were filtered based on Exome Aggregation Consortium (ExAC) allele filter thresholds for the general population [63]. Variants were prioritized on co-segregation patterns in pedigrees and absence of variants in control individuals within the dataset for which echocardiograms and ECGs were available. Cumulative sums of co-segregating rdSNVs were computed for all protein coding genes based on RefSeq gene annotation and transformed to z-scores. These counts were summed for 10 equal sized successive bins for the Residual Variation Intolerance Scores based on the ExAC population dataset. Significance was assessed under the null hypothesis that there should be no significant relationship between gene-based rdSNVs count and RVI scores (RVIS). In the presence of co-segregating rare mutations in genes that are intolerant or tolerant to mutations, larger counts in the low or high RVI score bins towards the left and right side of the distribution were tested for enrichment using the Wilcoxon test statistic. We further assessed the departure from the null hypothesis by evaluating the significance of the cumulative distribution by transforming z-scores to p-values. Adapted gene set enrichment analysis was performed on z-score based gene ranking for co-segregating rdSNVs with the weighted parameter and 1000 permutations against 50 hallmark gene sets from the Molecular Signatures Database [28,29]. Population stratification and IBD analysis was performed for whole-exome and target enrichment variant calls in order to exclude sample duplications and mismatches within family pedigrees. Principal components analysis on joined variant calls from 1000 genomes, study subjects and control samples from the Mendel cohort was performed using the SNPRelate package (version 1.0.1). Common variants (MAF > 5%). for 2504 individuals from the 1000 Genomes dataset phase 3 release and study subjects were plotted in the R software environment. All variants were LD pruned in SNPrelate with an LD threshold of 0.2 to produce a set of SNPs in approximate linkage equilibrium. PCA plots using the first two principal components were created using the ggplot2 package in R [64]. Prediction on variant deleteriousness for SIFT, Poly-Phen-2, Mutation Taster, Mutation Assessor and CADD scores were obtained through the ANNOVAR [65] annotation toolset for RefSeq gene annotation models.

### Variant validation

All prioritized variants for the presented families were validated using PCR-based bidirectional Sanger sequencing using BigDye 3.1 chemistry on an ABI 3130xl genetic analyzer (Applied Biosystems) to confirm mutations detected by whole exome sequencing. PCRs for family members were performed using primer pairs in S4 Table. Electropherograms were aligned to the NCBI build 37 reference sequence of the respective candidate locus and analysed using SeqMan Pro, which is part of the Lasergene suite (DNASTAR).

### Linkage analysis

DNA from whole blood from drawn from family 3 was used for genotyping on the Infinium OmniExpressExome-8 Bead Chip in order to validate the whole-exome sequencing association with *NOTCH1*. Genotypes were called with 99.8% accuracy. Raw genotypes were filtered using plink [66]. LD based pruning was applied using a window size of 50 SNPs and a total of 24551 markers were used for linkage analysis using Superlink Online [67]. Multipoint analysis was performed in Superlink using an autosomal-dominant mode of inheritance with a Disease Frequency of 0.01 and a MOI of 0, 0.99, 0.99 (S2 Fig). Results from linkage analysis were extracted from Superlink web-based output and subsequently visualized in Rstudio using the ggplot2 package [64].

### Web resources

The URLs for data presented herein are as follows:

1000Genomes, www.browser.1000genomes.org

RVIS, www.genic-intolerance.org/download.jsp

ANNOVAR, www.annovar.openbioinformatics.org

dbSNP, www.cbi.nlm.nih.gov./projects/SNP

Exome Aggregation Consortium (ExAC), www.exac.broadinstitute.org

Gene Set Enrichment analysis (GSEA) tool, www.broadinstitute.org/gsea

Broad derived hallmark gene sets, www.broadinstitute.org/gsea/msigdb/collection

Combined Annotation Dependent Depletion scores, www.cadd.gs.washington.edu

## Supporting Information

S1 Fig Candidate gene filtering in family based whole exome sequencing. Boxplots comparing distribution of remaining deleterious (missense and nonsense) variants in whole-exomes of patients for different ExAC frequency filtering thresholds. Co-segregation in multiple affected family members significantly reduced (p < 0.01, Wilcoxon) potential candidate variants independent of allele filtering thresholds
(TIF)

S2 Fig Excess of deleterious variants in highly conserved genes. Excess of rdSNVs along the upper and lower 10^th^ percentile of the genome-wide RVI score scale (p < 0.01, Wilcoxon) for rare variants (ExAC MF < 0.1%).
(TIF)

S3 Fig Candidate genes retained from whole-exome sequencing. Candidate genes retained after whole-exome variant filtering and gene prioritization based on co-segregation and pathway analysis. Candidate genes are grouped in three broad functional categories based on pathway and literature association.
(TIF)

S4 Fig Genome wide linkage analysis results for Family 3. **(A)** Each panel represents an autosomal chromosome. LOD scores above 1.5 are colored red. LOD = log-of-odds, cM = centimorgan. **(B)** List of all rare (ExAC MAF < 0.1%), deleterious variants co-segregating with TOF from 3 whole-exome sequenced probands (Family 3: III-4, IV-2, V-1) across three generations. The linkage peak on chromosome 9 (chr9:137352393–140964937) overlaps with co-segregating pathogenic mutations in *NOTCH1*.
(TIF)

S5 Fig Pedigree of Family 4 with co-segregating ARHGAP31 mutation. **(A)** Pedigree of family 4 harboring a protein truncating mutation in *ARHGAP31*. Colors represent the different phenotype associations. The (+/-) symbols indicate mutation carrier status. **(B)** Clinical features of patients carrying the stop-gain mutation (c.A4222T:p.K1408*) in *ARHGAP31*.
(TIF)

S6 Fig *ARHGAP31* expression pattern during embryo development in *Gallus gallus*. Each panel represents in situ hybridization pictures for *ARHGAP31* for different stages (9, 15, 19) in the course of chicken development obtained from the online repository GEISHA (www.geisha.arizona.edu) The expression pattern observed in chicken corresponds to a strong and specific expression of ARHGAP31 in the developing heart and vasculature. This strong expression pattern overlaps with *ARHGAP31* expression in the developing mouse heart as reported by Southgate et al [40].
(TIF)

S7 Fig Genetic Heterogeneity in candidate genes. **(A)** Principal component analysis of candidate genes against the ExAC dataset for rare stop-gain (PC1) and missense variants (PC2). Genes which have acquired a higher frequency of rare variants such as *KMT2D* (MLL2), *EP400* and *MYH6* cluster outside the majority of genes. **(B)** The ClinVar annotation [68] of candidate loci reflects the distribution of rare deleterious and benign mutations in genes which have acquired an excess of rare variants.
(TIF)

S8 Fig Pedigree of Family 5 with compound heterozygous *MYH6* mutations. **(A)** Pedigree of family 5 harboring a compound heterozygous mutation in *MYH6*. Notably, the paternal variant p.M494I shows incomplete penetrance at II-2 and leads in combination with the maternal variant p.E1885K to a more severe phenotype (Hypoplastic left heart). Colors represent the different phenotype associations. The (+/-) symbols indicate mutation carrier status. **(B)** Clinical features of *MYH6* mutation carriers.
(TIF)

S1 Table Excess of rare deleterious variants in highly conserved genes. Genes among the top 10% RVIs genome-wide scale are highlighted harboring an excess of rare (ExAC < 0.1%) deleterious variants (>10 rdSNVs). Z-scores are calculated based on variant distribution for 106 affected individuals and transformed in p-values.
(XLS)

S2 Table Whole-exome filtering strategies for the identification of pathogenic variants in Families 1–4. The tables depict the individual filtering strategies for Families 1–3 with novel pathogenic protein disrupting mutations in *NOTCH1* and *ARHGAP31* (Family 4). Filtering was performed based on allele frequency cutoffs (ExAC MF < 0.1%) and segregation with disease among multiple affected family members.
(XLS)

S3 Table CADD ranked variant pathogenicity in candidate genes. Co-segregating, private variants sorted based on scaled CADD score pathogenicity in targeted re-sequenced candidate genes.
(XLS)

S4 Table Variant pathogenicity in Broad derived hallmark gene sets. The table lists the excess of pathogenicity based on scaled CADD scores in affected individuals for candidate loci. Significance was determined by comparing variants in affected individuals and unrelated and unaffected parents using Wilcoxon rank-sum tests with subsequent FDR p-value correction for multiple testing.
(XLS)

S5 Table Primer pairs used for Sanger Sequencing Validation. Primers used for Sanger sequencing validation of rare deleterious and co-segregating SNVs in the study.
(XLS)
