# Supplementary figures and images for: Family Based Whole Exome Sequencing Reveals the Multifaceted Role of Notch Signaling in Congenital Heart Disease
Source: PLoS Genet. 2016 Oct 19;12(10):e1006335. doi: 10.1371/journal.pgen.1006335 (PMC5070860; doi:10.1371/journal.pgen.1006335)

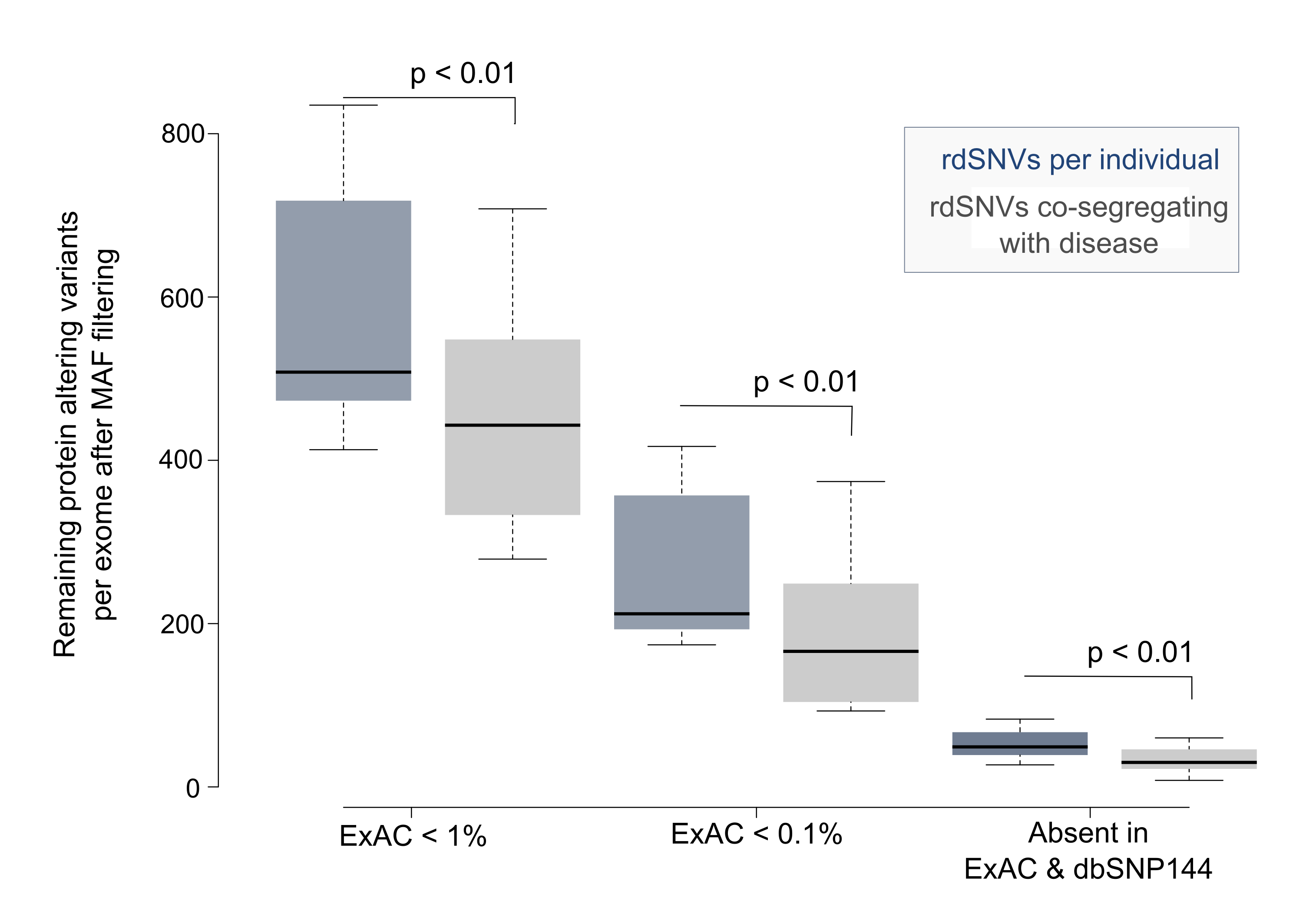

Supplement: S1 Fig — Boxplots comparing distribution of remaining deleterious (missense and nonsense) variants in whole-exomes of patients for different ExAC frequency filtering thresholds. Co-segregation in multiple affected family members significantly reduced (p < 0.01, Wilcoxon) potential candidate variants independent of allele filtering thresholds (TIF) [file pgen.1006335.s001.tif]

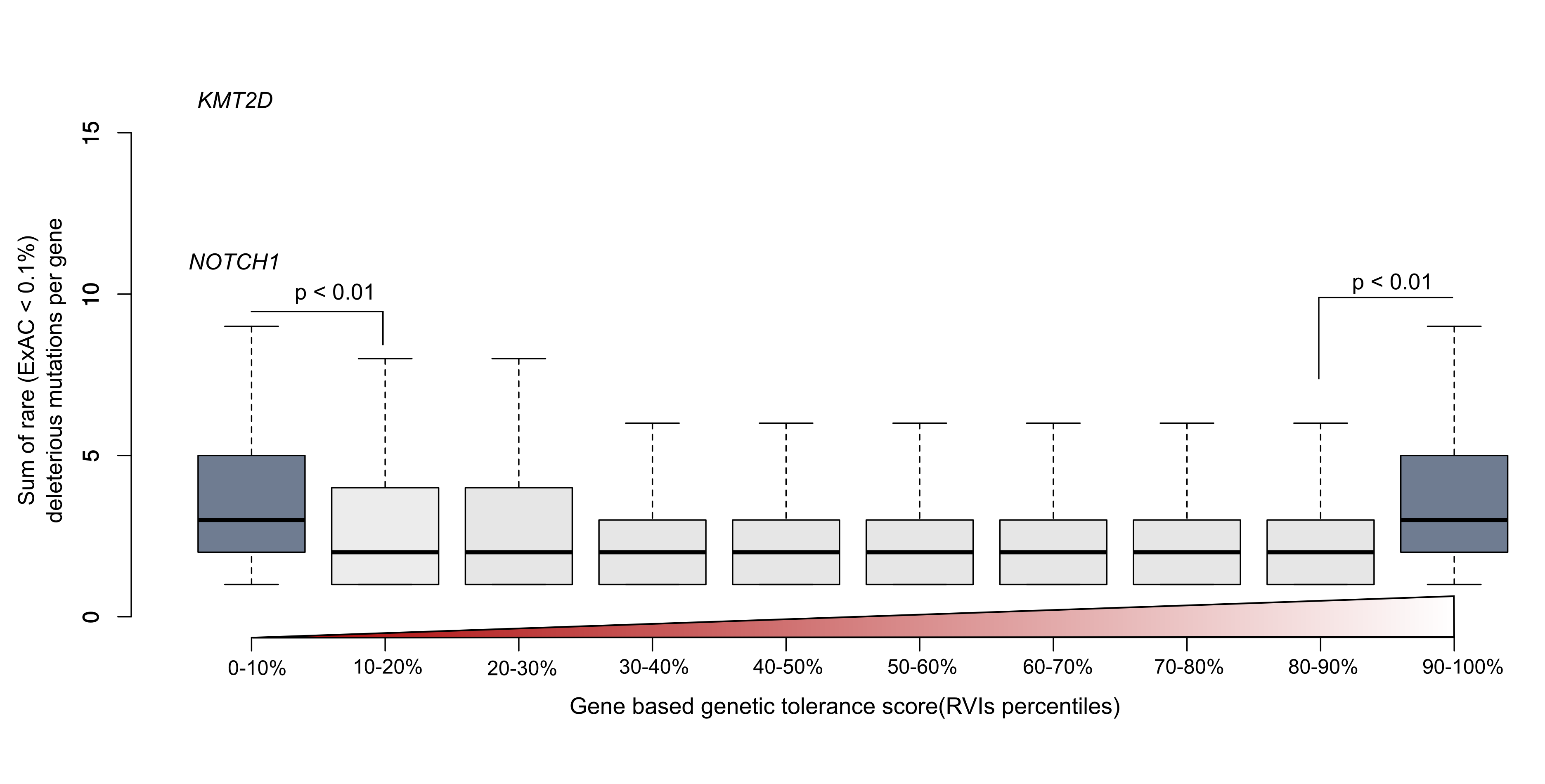

Supplement: S2 Fig — Excess of rdSNVs along the upper and lower 10th percentile of the genome-wide RVI score scale (p < 0.01, Wilcoxon) for rare variants (ExAC MF < 0.1%). (TIF) [file pgen.1006335.s002.tif]

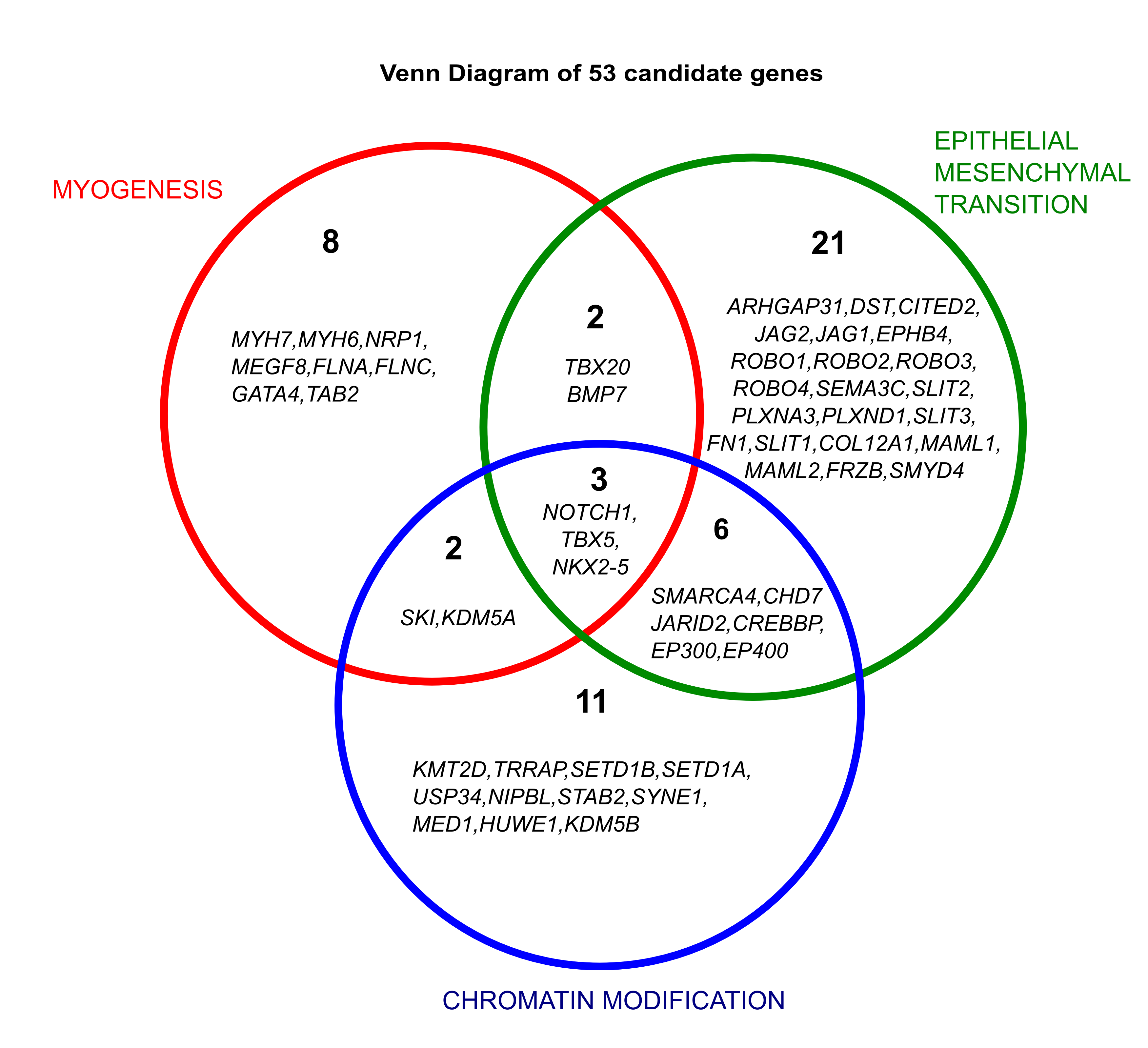

Supplement: S3 Fig — Candidate genes retained after whole-exome variant filtering and gene prioritization based on co-segregation and pathway analysis. Candidate genes are grouped in three broad functional categories based on pathway and literature association. (TIF) [file pgen.1006335.s003.tif]

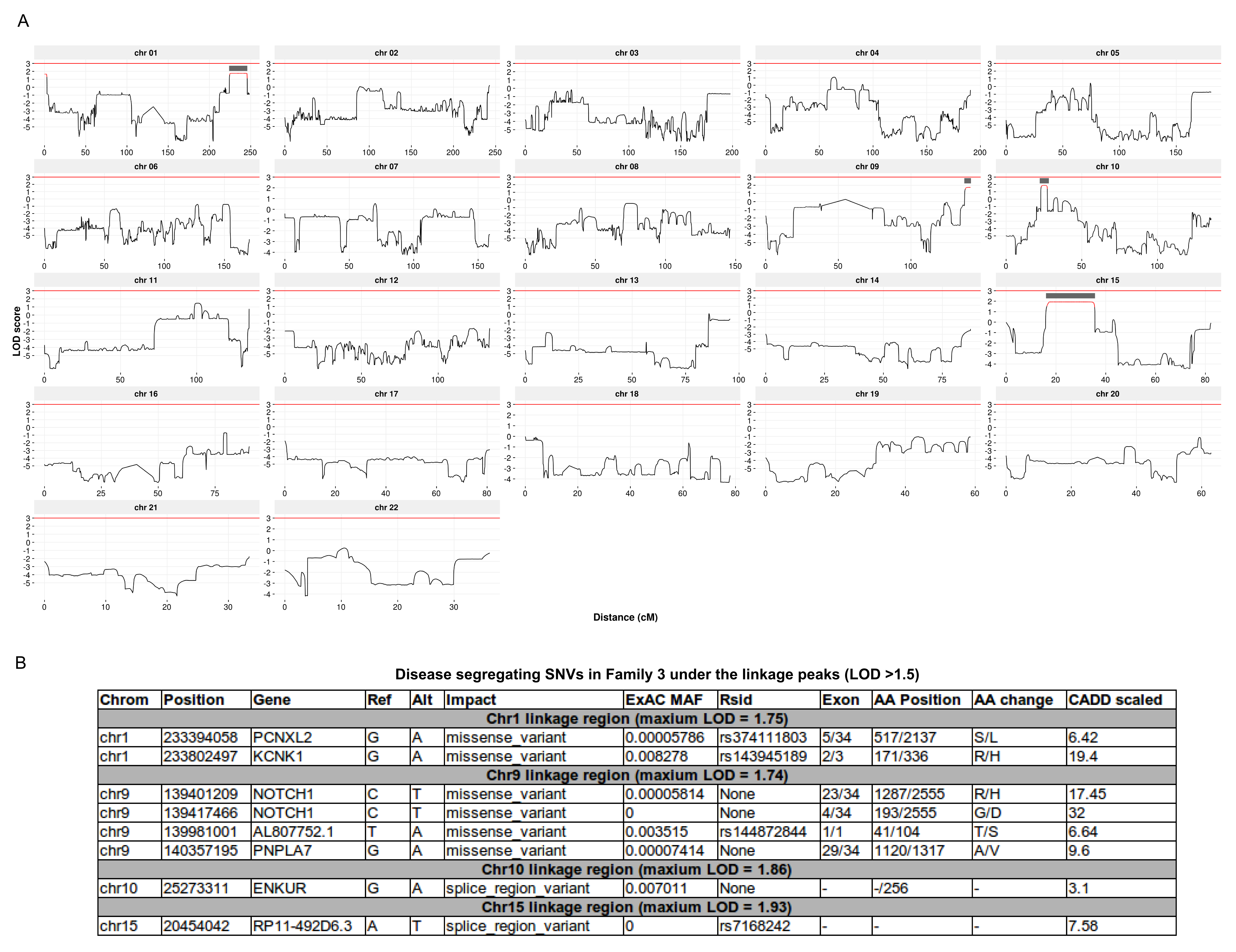

Supplement: S4 Fig — (A) Each panel represents an autosomal chromosome. LOD scores above 1.5 are colored red. LOD = log-of-odds, cM = centimorgan. (B) List of all rare (ExAC MAF < 0.1%), deleterious variants co-segregating with TOF from 3 whole-exome sequenced probands (Family 3: III-4, IV-2, V-1) across three generations. The linkage peak on chromosome 9 (chr9:137352393–140964937) overlaps with co-segregating pathogenic mutations in NOTCH1. (TIF) [file pgen.1006335.s004.tif]

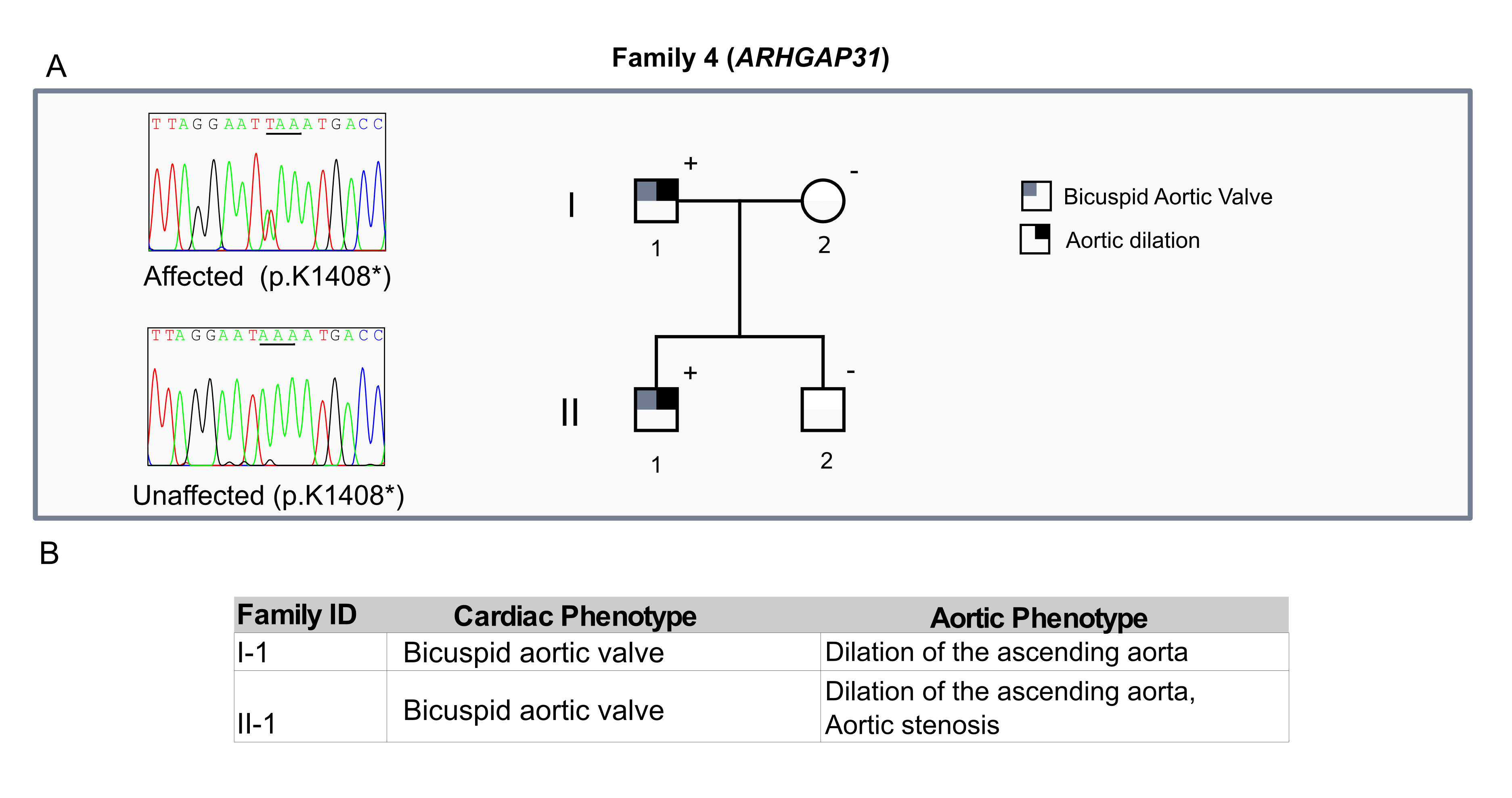

Supplement: S5 Fig — (A) Pedigree of family 4 harboring a protein truncating mutation in ARHGAP31. Colors represent the different phenotype associations. The (+/-) symbols indicate mutation carrier status. (B) Clinical features of patients carrying the stop-gain mutation (c.A4222T:p.K1408*) in ARHGAP31. (TIF) [file pgen.1006335.s005.tif]

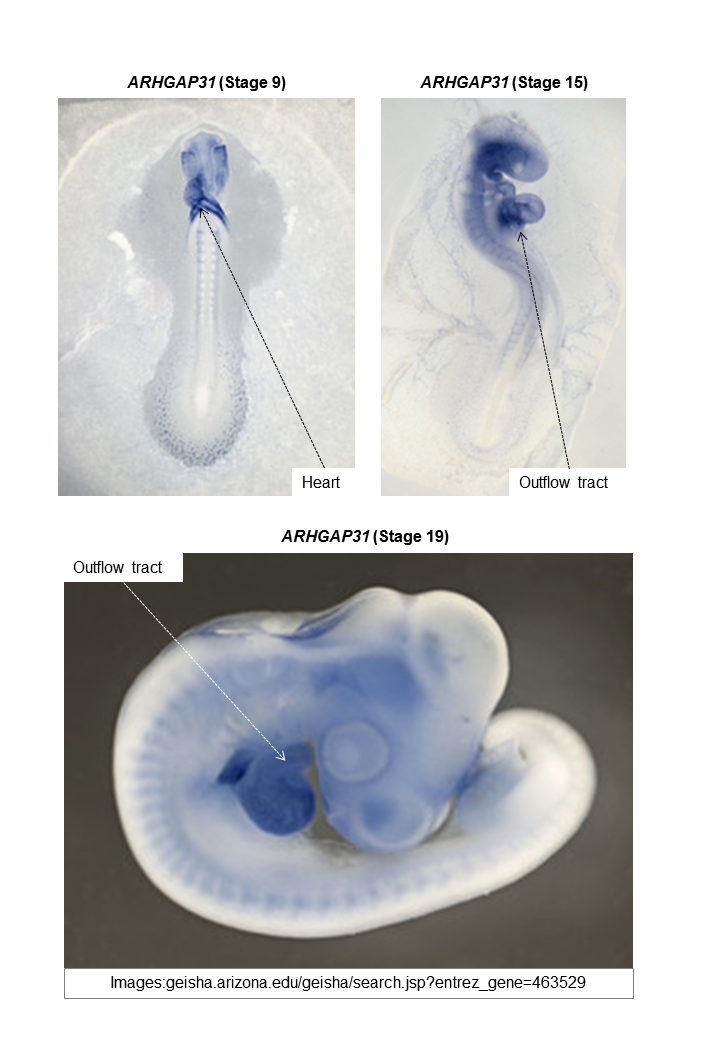

Supplement: S6 Fig — Each panel represents in situ hybridization pictures for ARHGAP31 for different stages (9, 15, 19) in the course of chicken development obtained from the online repository GEISHA (www.geisha.arizona.edu) The expression pattern observed in chicken corresponds to a strong and specific expression of ARHGAP31 in the developing heart and vasculature. This strong expression pattern overlaps with ARHGAP31 expression in the developing mouse heart as reported by Southgate et al [40]. (TIF) [file pgen.1006335.s006.tif]

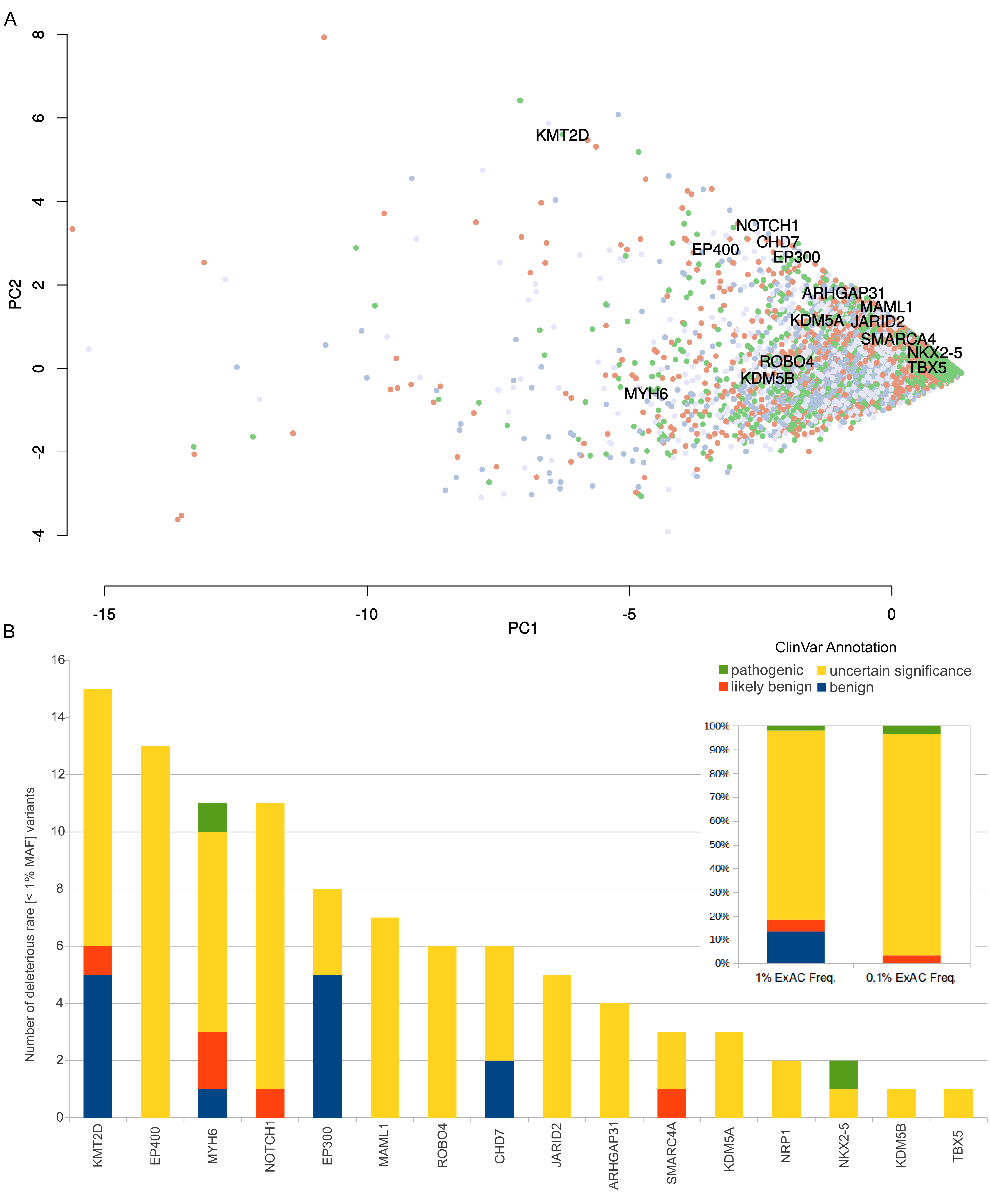

Supplement: S7 Fig — (A) Principal component analysis of candidate genes against the ExAC dataset for rare stop-gain (PC1) and missense variants (PC2). Genes which have acquired a higher frequency of rare variants such as KMT2D (MLL2), EP400 and MYH6 cluster outside the majority of genes. (B) The ClinVar annotation [68] of candidate loci reflects the distribution of rare deleterious and benign mutations in genes which have acquired an excess of rare variants. (TIF) [file pgen.1006335.s007.tif]

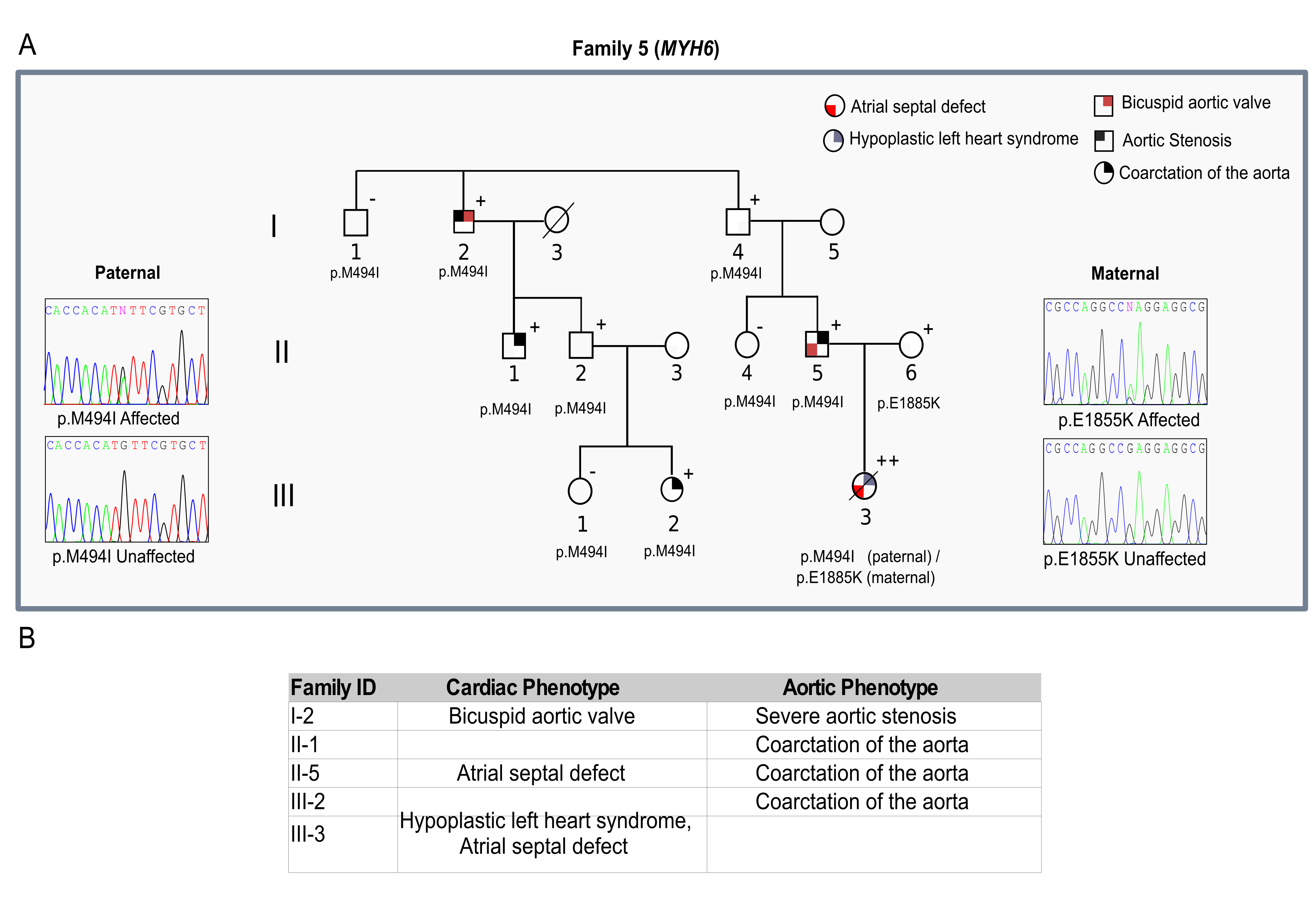

Supplement: S8 Fig — (A) Pedigree of family 5 harboring a compound heterozygous mutation in MYH6. Notably, the paternal variant p.M494I shows incomplete penetrance at II-2 and leads in combination with the maternal variant p.E1885K to a more severe phenotype (Hypoplastic left heart). Colors represent the different phenotype associations. The (+/-) symbols indicate mutation carrier status. (B) Clinical features of MYH6 mutation carriers. (TIF) [file pgen.1006335.s008.tif]
